# Drivers of hospital expenditure and length of stay in an academic medical centre: a retrospective cross-sectional study

**DOI:** 10.1186/s12913-019-4248-1

**Published:** 2019-07-02

**Authors:** Nabilah Rahman, Sheryl Hui-Xian Ng, Sravan Ramachandran, Debby D. Wang, Srinath Sridharan, Chuen Seng Tan, Astrid Khoo, Xin Quan Tan

**Affiliations:** 1 Centre for Health Services and Policy Research, Saw Swee Hock School of Public Health, National University of Singapore and National University Health System, 12 Science Drive 2, Singapore, Singapore; 2 Saw Swee Hock School of Public Health, National University of Singapore and National University Health System, 12 Science Drive 2, Singapore, Singapore; 3 Regional Health System Planning Office, National University Health System, 1E Kent Ridge Road, Singapore, Singapore

**Keywords:** Electronic medical records, Utilization, Health service, Hospital resources

## Abstract

**Background:**

As healthcare expenditure and utilization continue to rise, understanding key drivers of hospital expenditure and utilization is crucial in policy development and service planning. This study aims to investigate micro drivers of hospital expenditure and length of stay (LOS) in an Academic Medical Centre.

**Methods:**

Data corresponding to 285,767 patients and 207,426 inpatient visits was extracted from electronic medical records of the National University of Hospital in Singapore between 2005 to 2013. Generalized linear models and generalized estimating equations were employed to build patient and inpatient visit models respectively. The patient models provide insight on the factors affecting overall expenditure and LOS, whereas the inpatient visit models provide insight on how expenditure and LOS accumulate longitudinally.

**Results:**

Although adjusted expenditure and LOS per inpatient visit were largely similar across socio-economic status (SES) groups, patients of lower SES groups accumulated greater expenditure and LOS over time due to more frequent visits. Admission to a ward class with greater government subsidies was associated with higher expenditure and LOS per inpatient visit. Inpatient death was also associated with higher expenditure per inpatient visit. Conditions that drove patient expenditure and LOS were largely similar, with mental illnesses affecting LOS to a larger extent. These observations on condition drivers largely held true at visit-level.

**Conclusions:**

The findings highlight the importance of distinguishing the drivers of patient expenditure and inpatient utilization at the patient-level from those at the visit-level. This allows better understanding of the drivers of healthcare utilization and how utilization accumulates longitudinally, important for health policy and service planning.

**Electronic supplementary material:**

The online version of this article (10.1186/s12913-019-4248-1) contains supplementary material, which is available to authorized users.

## Background

Globally, healthcare expenditure and utilization are increasing at unsustainable rates. Based on a recent study, global expenditure on health is expected to almost triple from 2014 to 2040 [1]. Health expenditure has risen faster than economic growth in Organisation for Economic Co-operation and Development (OECD) countries [2]. Many countries have also reported shortage in healthcare resources due to an increased demand for healthcare services [3–5]. Similarly, Singapore is experiencing a surge in healthcare expenditure, worsened by manpower and infrastructure challenges [5–8]. Singapore healthcare spending has doubled from $4 billion in 2011 to $9.8 billion in 2016 [9, 10], to become the third largest expenditure item [11]. Hospital expenditure from provision of acute care accounts for the majority of Singapore’s overall ongoing healthcare expenditure [12]. High hospital utilization has resulted in significant investment to increase capacity [13], and is of concern to policy makers.

Healthcare expenditure and utilization can be studied at a macro or micro level. At macro level, measures of interest will be studied by state or/and year [14–17], and common explanatory variables are gross domestic product (GDP) indices [14–17], age distribution [14–17], health indicators and supply factors [14, 15, 17]. These analyses are routinely done and are useful for understanding performance at a health system level. At micro level, measures of interest are studied by patient or visit [18–21]. These analyses are critical in understanding patient and condition drivers of expenditure and utilization, allowing healthcare planners and professionals to better design policies and programs at the meso level in a data driven manner. While there have been several studies looking at micro factors within specific clinical subpopulations driving the increase in expenditure and utilization [18–21], such analyses on general population are scarce [22], resulting in a knowledge gap on general patient and visit factors affecting expenditure and utilization. To the best of our knowledge, there are currently only four published studies examining micro factors in the general population. The first is an early study in the United States of America (USA) where total hospital expenditure among full-year Medicaid enrollees was regressed on length of stay (LOS), surgery use and location of medical care services [23]. LOS of these enrollees was modeled with primary diagnosis (PD), death indicator, socio-economic status (SES), number of days in bed and location of utilization as explanatory variables. The other study in Tajikistan examined out-of-pocket (OOP) inpatient expenditure and LOS [24]. It highlighted SES, chronic status, surgery, intensive care and cancer as main factors which explained inpatient expenditure. Chronic status of patients, disease type (i.e. tuberculosis and hepatitis), treatment type and hospital type were important factors for LOS. Another study examined annual medical expenditure of rural residents in China using three-level linear model and highlighted age, disease category, inpatient status, healthcare utilization and utilization level as drivers of annual medical expenditure [25]. The last study used longitudinal analyses to study the effects of age and time to death on hospital expenditure [26].

Results from the above-mentioned studies have been useful in helping the healthcare community better understand the key drivers of hospital expenditure and utilization, for development of policies and service planning. Given the lack of recent studies done at the micro level, this study aims to address these knowledge gaps through exploring the drivers of expenditure and LOS at both the patient- and visit-level, in the general subsidized adult population of an Academic Medical Centre (AMC) in Singapore. Impact of these drivers on patient expenditure and LOS will be compared, and differences at the patient-level and visit-level will be examined to provide additional insight on the variation in drivers of increased expenditure and utilization.

## Methods

### Study samples

This is a retrospective cross-sectional study of subsidized patients in National University Hospital (NUH), a 1000-bed AMC in National University Health System (NUHS) Singapore. Being one of two AMCs in Singapore, an urban city-state with a multi-ethnic population of 5.6 million, this study gives an important overview of the drivers of hospital expenditure and LOS in a tertiary care setting, and developed nation in Asia. Based off life expectancy and corresponding health expenditure, the city-state has been consistently rated to have one of the most efficient healthcare system in the world [27]. With a life expectancy of 82.7 years in 2015, the relative healthcare expenditure accounted for only 4.3% of total GDP, and per capita absolute healthcare expenditure stood at US $2752. These are in comparison to OECD average life expectancy of 80.6 years in 2015, relative healthcare expenditure accounting for 9% of total GDP and per capita absolute healthcare expenditure of US $4003 [28].

Access to data from the NUH’s electronic medical record (EMR) for the period of 2005–2013 was granted in 2016 for this study where 2005 was the first full year that the EMR system had been implemented. Data up to 2018 was not available for analysis due to the time required to seek for data access, de-identify the data to protect patient privacy, and pre-process the data to facilitate research. Between 2005 and 2013, the system recorded a total of 10,795,573 inpatient and outpatient visits. An increasing number annual visits was observed during the period [29]. Both patient- and visit-level data were studied. Analyzing patient-level data provides insight on the factors affecting overall utilization for a patient, including determinants such as demographic factors and SES, as well as overall health status. Studying utilization at the visit-level is complementary, providing an opportunity to study more directly the impact of condition on expenditure and LOS, and augments our understanding of how expenditure and LOS accumulate longitudinally. Only inpatient visits were considered for visit-level data.

The framework and pipeline to generate a base cohort of 549,109 adult patients and 411,266 inpatient visits from raw EMR records have been documented previously [29]. Exclusion criteria were further applied to the adult patient cohort, resulting in 285,767 patients in our sample. The exclusion criteria applied at patient-level involved:

1. Receipt of unsubsidized care. This was to restrict our analyses to only subsidized patients due to differences in expenditure computation and based on policy relevance.
2. No mapped PD by Clinical Classification Software (CCS) [30]. This ensured that patients had at least a valid history of condition which the patient sought care for.
3. Utilization after recorded death date and zero expenditure despite utilization. These patients were excluded due to potential incongruence in records.
4. Missing resident status and housing type (SES proxy). These ensured that socio-demographic information was available for each patient.

These remaining 285,767 patients accounted for 213,425 inpatient visits. Thereafter, exclusion criteria were applied to the 213,425 inpatient visits to give 207,426 inpatient visits. The exclusion criteria applied at visit-level involved:

1. Inpatient visits with missing CCS PD, which was needed in the analyses.
2. Missing ward class, information which was also needed in the analyses.
3. Zero expenditure despite the non-zero LOS. These visits were excluded due to potential incongruence in records.

**Fig. 1:**
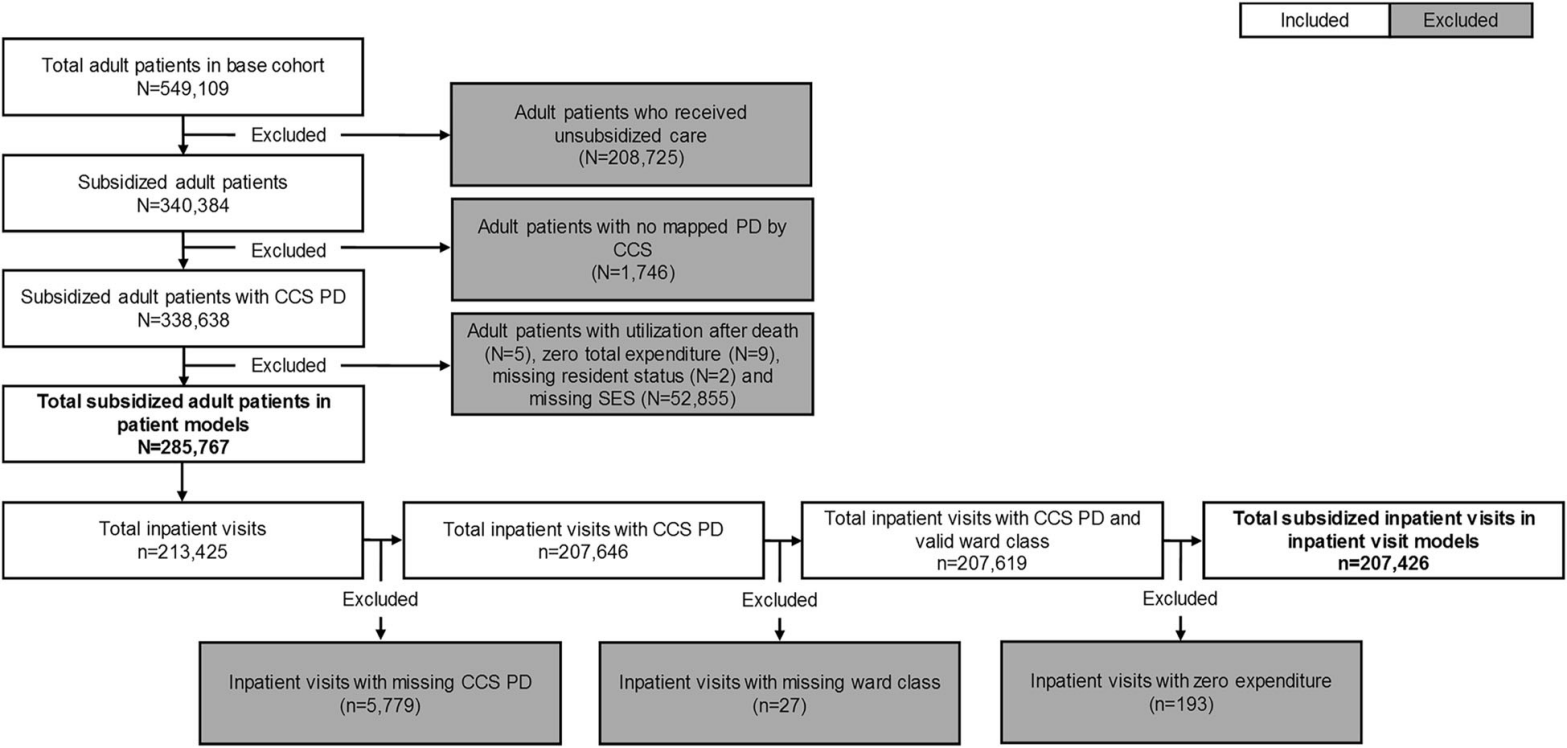
Flowchart of exclusion criteria applied to obtain study samples

The flowchart of study sample construction is depicted in Fig. 1.

### Outcome variables

The four outcome variables of interest were expenditure per patient (expenditure (P)), LOS per patient (LOS (P)), expenditure per inpatient visit (expenditure (V)) and LOS per inpatient visit (LOS (V)). Expenditure refers to the bill size of the hospital services before subsidy, adjusted to 2015 Singapore dollars. LOS computation in this study excluded hospitalization that is attributed to day procedures (e.g. day surgery and endoscopy), similar to the methodology adopted by OECD studies [31]. Expenditure (P) and LOS (P) refer to the total expenditure and LOS over observed period for each patient. Expenditure (V) and LOS (V) refer to the expenditure and LOS for that specific inpatient visit.

### Explanatory variables

The following patient-level explanatory variables were included in our study: (1) demographics (gender, ethnicity, age group, housing type, resident status), (2) frequent PD, and (3) observed period. Age group was derived from age as at first contact during the study period. Housing in Singapore can be divided into three main types: private housing, public housing, and public rental housing. Private housing caters mainly to the upper-middle- to upper-income groups, public housing caters to the middle-income group, whereas public rental housing serves as social housing for the low-income group in Singapore. In 2013, approximately 78% of the citizens and permanent residents (PRs) resided in public housing as owner-occupiers, whereas 19% resided in private housing and 3% resided in public rental housing [32, 33]. Rental, direct purchases and grants of public housing are restricted to resident households with monthly income not exceeding pre-specified income ceilings [34]. For instance, the public rental units are reserved for households with monthly income not exceeding SG $1500 and direct purchases of public housing are restricted to households with monthly income not exceeding SG $12,000 [34]. Due to its close association with income, housing type was used as a proxy for SES. The use of housing type as a proxy for SES has also been validated in an earlier study [29]. The study showed that larger and private housing types were associated with lower government subsidies received. As patients from higher income groups receive lesser subsidies, this also suggests that larger and private housing types are associated with higher SES. The housing types in ascending order of SES level are as follows: Rental, studios, 1–2-room, 3-room, 4-room, 5-room and private. It was based on the most recent housing information. Resident status refers to whether a patient was a Singaporean or PR. Patients were coded to have a particular PD if the particular CCS condition had ever been the main condition they sought care for during the study period [30]. Frequent PD examined in this study were conditions that were either (1) one of the five most frequent inpatient or outpatient PD in patients who are in the top 10% of expenditure (P) and LOS (P) of the base cohort, or (2) PD included in the Charlson Comorbidity Index (CCI) and had at least 1500 patients diagnosed with the PD in the base cohort [35]. Cutoffs were incorporated in these criteria to ensure that there were sufficient numbers for analysis and to limit the number of conditions for interpretability of the model. Observed period was the number of years between date of first contact and end of study period or death date and was included to account for duration of follow-up. Given the abundant sample size, expenditure (P) and LOS (P) were regressed on all these patient factors to give patient models. These models were built to examine the association of patient factors with expenditure (P) and LOS (P) respectively.

The following visit-level explanatory variables were included in our study: (1) demographics (gender, ethnicity, age group, housing type, resident status), (2) PD, and (3) Others (ward class, CCI and inpatient death). Age group was derived from age at the point of visit. PD included in the model were the top ten PD with the largest effect size in the respective patient models, ‘sprains and strains’ (as a reference condition) and ‘others’. ‘Sprains and strains’ was chosen as the reference condition given their relatively high prevalence while low in expenditure and likelihood of requiring intensive treatment. The ‘others’ group was a heterogeneous group consisting of all other PD not represented. In Singapore, the level of subsidy a citizen receives is based on his ability to pay (determined through means testing) and the ward class (i.e. ward A, B1, B2 and C in order of increasing government subsidies and decreasing OOP expenses assuming the same services consumed) [36]. As patients are allowed to change their ward class during their inpatient stay, we used the last class of ward that the patients stayed in during their visits as the ward class. We have only two categories of ward classes in our data as we are looking at only subsidized patients. Patients staying in B2 class wards receive 50 to 65% subsidies whereas patients staying in C class wards receive 65 to 80% subsidies [37]. Given that two patients received the exact same care, the patient staying in the ward with higher subsidy would have lower OOP expenses. CCI was calculated for each visit using past medical condition history. Similarly, expenditure (V) and LOS (V) were regressed on all these visit factors to give inpatient visit models. These models were built to examine the association of visit factors with expenditure (V) and LOS (V) respectively.

Utilization factors such as LOS, Intensive Care Unit (ICU) days, Specialist Outpatient Clinic (SOC) visits and Emergency Department (ED) visits were excluded from the set of explanatory variables for the expenditure models because they are a direct function of hospital expenditure and its association with expenditure would reflect the pricing mechanism. Moreover, these variables are likely to exhibit endogeneity with the non-utilization explanatory variables in the expenditure models. Similarly, utilization factors such as ICU days, SOC visits and ED visits were excluded from the set of explanatory variables for the LOS models as they are likely to exhibit endogeneity with the non-utilization explanatory variables in the LOS models.

### Statistical analyses

As expenditure (P) and LOS (P) exhibited skewed distribution, rendering traditional ordinary least square model inappropriate, generalized linear models (GLM) were used to model these per patient outcome variables. Modified Park’s test [38], which tests the specific form of heteroscedasticity, was used to identify whether gamma GLM was a suitable family for expenditure (P) model [38]. Negative binomial GLM was used to model LOS (P) as it models count data as well as over-dispersion. Multicollinearity was checked using degrees of freedom corrected generalized variance-inflation factor (GVIF) [39]. GVIF is a measure to check for multicollinearity in regression models with categorical variables. Smaller GVIF value is preferred. Correcting GVIF using degrees of freedom allows GVIF to be comparable across dimensions. Degrees of freedom corrected GVIF of below 3.15 (equivalent to variance-inflation factor value of below 10) indicates inconsequential collinearity [40]. Generalized estimating equations (GEE) were used to account for the dependence of repeated outcomes from multiple inpatient visits of the same patient. It allows the estimation of the average effect of the visit-level explanatory variables on a specific inpatient visit [41, 42]. Intra-patient dependence of utilization was assumed to have a first-order autoregressive covariance structure. GEE with gamma and poisson families were used to model expenditure (V) and LOS (V) respectively. Sandwich variance estimator was used as it produces robust standard error when covariance structure is misspecified [43]. Log was used as link function for all the GLM and GEE models.

Post-hoc and subgroup analyses were also performed to better understand findings from the above analyses. The post-hoc and subgroup analyses consisted of correlation analysis and simple descriptive analysis using summary statistics. Statistical significance was assessed using a threshold of 0.01. RStudio Version 1.1.4 was used to perform the analyses [44]. The R package ‘geepack’, v. 1.2–0 [45] was used to build the GEE models.

## Results

### Baseline characteristics

The baseline characteristics of the 285,767 patients and 207,426 inpatient visits included in the study are described in Table 1 and Table 2. Majority of the patients were Singaporean (85%), male (57%), Chinese (65%), between 21 and 29 years at first contact during the study period (27%) and lived in a 4-room public flat (35%). Majority of the visits were by Singaporeans (95%), males (53%), Chinese patients (63%), 70–79 years (19%) and patients who lived in 4-room public flat (36%). About 3% of the inpatient visits ended in death.

**Table 1:** Summary of categorical characteristics of patients and inpatient visits that were included in the study

Categorical variable	Frequency (%)	
	By patient (N = 285,767)	By inpatient visit (n = 207,426)
Female	124,119 (43.4)	97,601 (47.1)
Ethnicity		
Chinese	186,202 (65.2)	131,574 (63.4)
Indian	26,275 (9.2)	19,277 (9.3)
Malay	48,928 (17.1)	42,519 (20.5)
Others	24,362 (8.5)	14,056 (6.8)
Age^a^		
21–29	77,860 (27.2)	18,395 (8.9)
30–39	48,857 (17.1)	16,744 (8.1)
40–49	48,635 (17.0)	25,013 (12.1)
50–59	48,276 (16.9)	39,369 (19.0)
60–69	30,823 (10.8)	38,148 (18.4)
70–79	20,781 (7.3)	39,983 (19.3)
80 and above	10,535 (3.7)	29,774 (14.4)
Housing type (SES proxy)		
Rental, studios, 1–2-room	12,382 (4.3)	15,523 (7.5)
3-room	66,071 (23.1)	58,189 (28.1)
4-room	99,944 (35.0)	74,342 (35.8)
5-room	78,118 (27.3)	49,883 (24.0)
Private	29,252 (10.2)	9489 (4.6)
Singaporean	243,112 (85.1)	197,096 (95.0)
Inpatient death	6546 (2.3)	6451 (3.1)

^a^Refers to age as at first contact for patient-level and age as at visit for visit-level

**Table 2:** Summary of numerical characteristics of patients and inpatient visits that were included in the study

Numerical variable	Total (median; interquartile range)	
	By patient (N = 285,767)	By inpatient visit (n = 207,426)
Hospital expenditure	2,341,299,199 (1275; 324–5945)	1,542,404,950 (3632; 1857–7859)
Length of stay	1,356,497 (0; 0–3)	1,310,109 (4; 2–7)
Inpatient visits	213,425 (0; 0–1)	
CCI	(0; 0–0)	(1; 0–4)
Observed period (years)	(5; 2–7)	

### Patient models

**Fig. 2:**
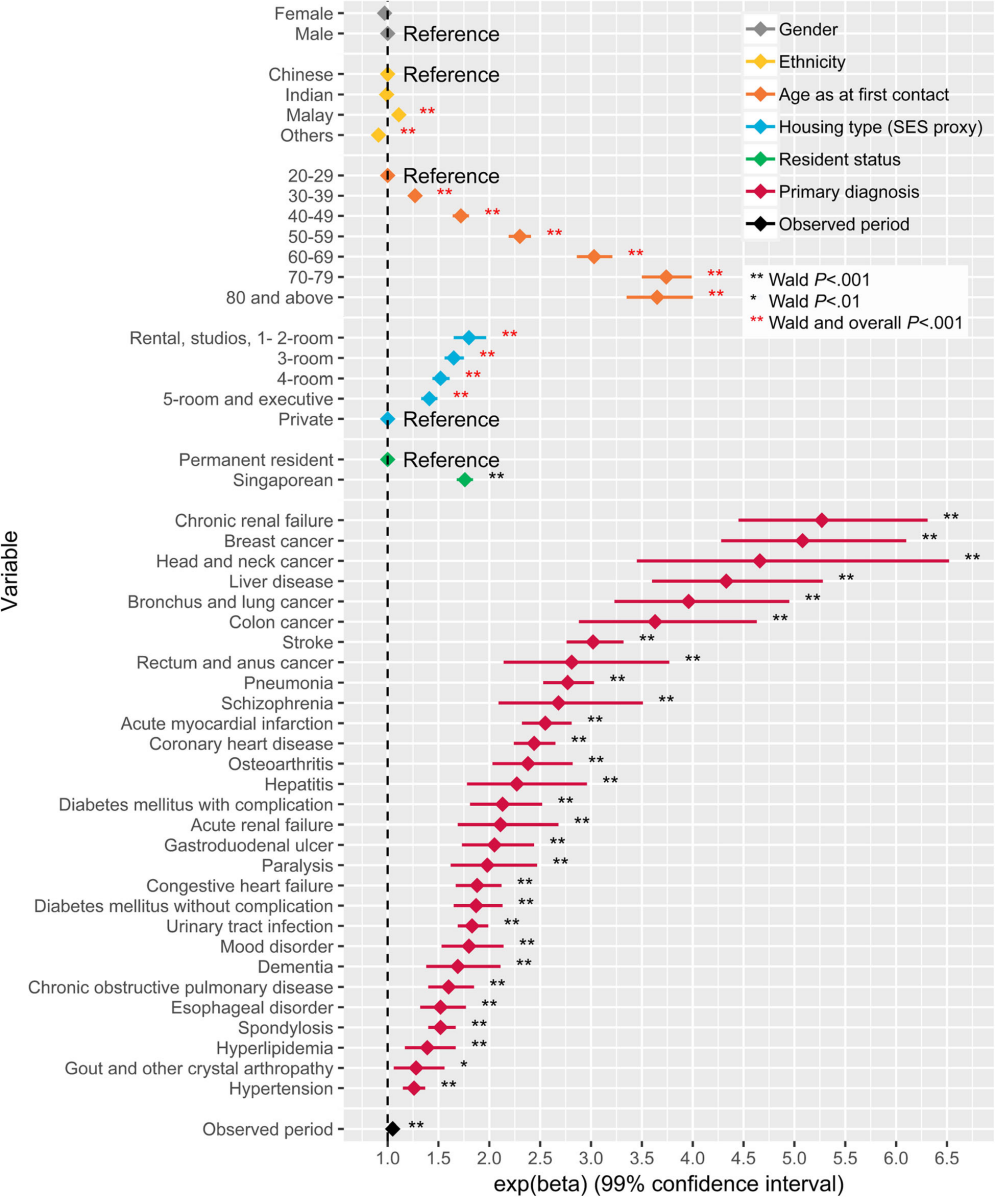
Visualization of the effects of patient factors on expenditure per patient (expenditure (P)). The diamond corresponds to the estimated effect and the horizontal line corresponds to the 99% confidence interval

Modified Park’s test indicated that the gamma family specification was adequate for expenditure (P) model. Degrees of freedom corrected GVIF values of the explanatory variables were all below 1.2, indicating inconsequential collinearity. The expenditure (P) model showed that ethnicity, age, housing type (SES proxy), resident status, PD and observed period were significantly associated with expenditure (P) with large dynamic ranges in their effects (Additional file 1). Large effects were observed for age, housing type, resident status and PD when compared with the other variables in the model. As compared to the youngest age group, patients in older age groups were expected to have higher expenditure (P) and this increase in expenditure plateaued at the oldest age group (Fig. 2). For instance, the expenditure (P) of the 30–39 age group was 27% (99% confidence interval (CI): 21–33%) higher when compared to the youngest age group. The effect monotonically increased and then peaked at the 70–79 age group where expenditure (P) was 274% (99% CI: 250–299%) higher than the youngest age group. The 80 and above group had 265% (99% CI: 235–300%) higher expenditure (P) when compared to the youngest age group. Compared to patients who lived in private housing, patients who lived in smaller public housing were expected to have higher expenditure (P), with the effect decreasing monotonically from smaller to bigger housing type (Fig. 2). For instance, patients who lived in 2-room or smaller public flats had 80% (99% CI: 65–97%) higher expenditure (P) than those who lived in private housing. Singaporeans had 76% (99% CI: 68–84%) higher expenditure (P) than PRs. Having the PD of chronic renal failure, breast cancer, head and neck cancer or liver disease at any point during the study period was associated with at least 300% higher expenditure (P) in comparison to when these PD were absent.

**Fig. 3:**
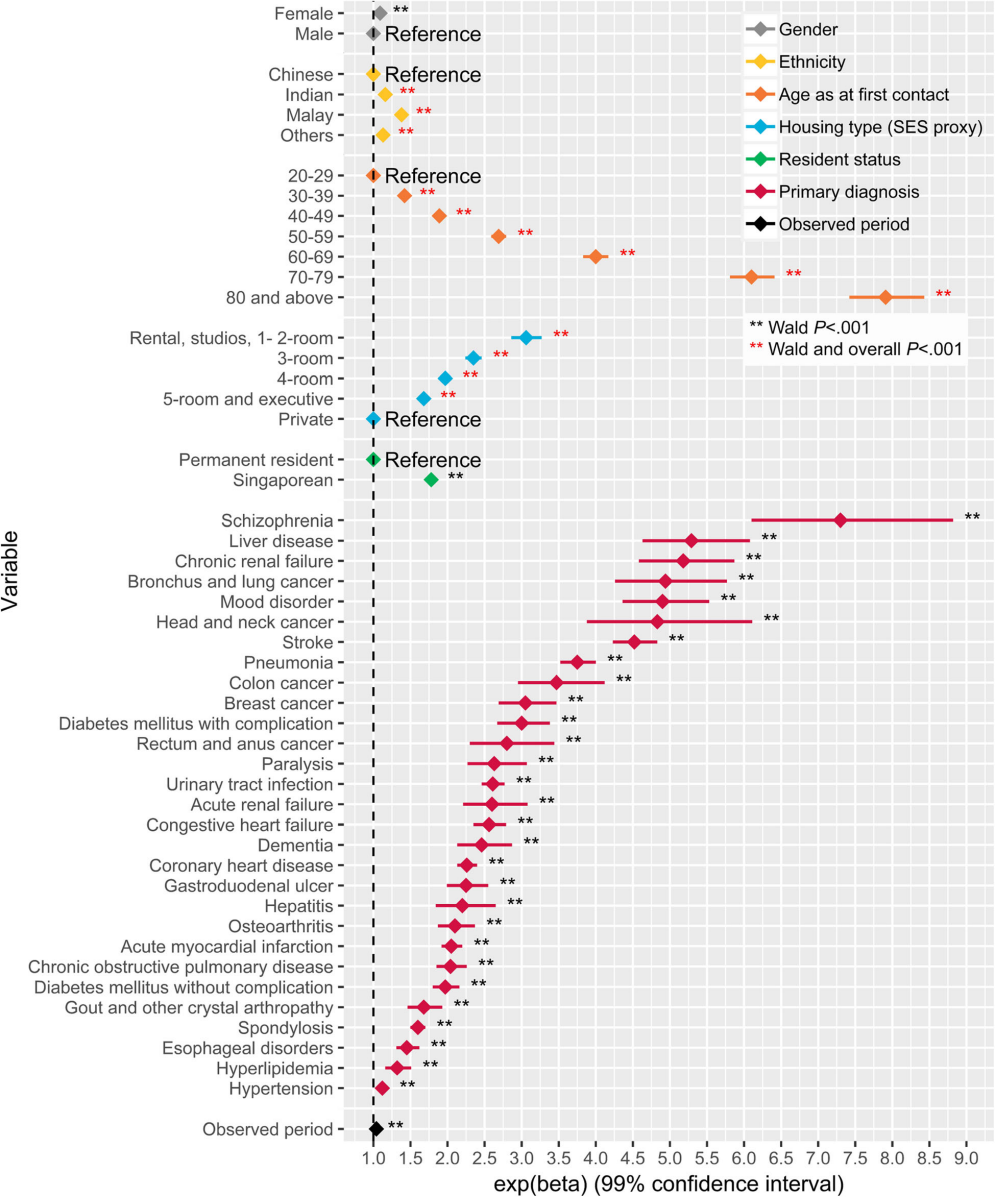
Visualization of the effects of patient factors on length of stay per patient (LOS (P)). The diamond corresponds to the estimated effect and the horizontal line corresponds to the 99% confidence interval

The LOS (P) model showed that gender, ethnicity, age, housing type, resident status, PD and observed period were significantly associated with LOS (P) with substantial variation in their effects (Additional file 2). Large effects were observed for ethnicity, age, housing type, resident status and PD when compared to other variables in the model. The Indians, Malays and patients of other ethnicities had 16% (99% CI: 11–20%), 38% (99% CI: 34–42%) and 13% (99% CI: 8–18%) higher LOS (P) when compared to the Chinese patients. When compared to the 21–29 age group, patients in older age groups were expected to have higher LOS (P), with age effect increasing monotonically from the youngest to the oldest age group (Fig. 3). The effect of housing type and resident status on LOS (P) were similar to the expenditure (P) model, albeit more pronounced here for housing type (Fig. 3). Having the PD of schizophrenia, liver disease, chronic renal failure, bronchus and lung cancer, mood disorder, head and neck cancer or stroke at any point during the study period was associated with at least 300% higher LOS (P) in comparison to when these PD were absent.

### Inpatient visit models

**Fig. 4:**
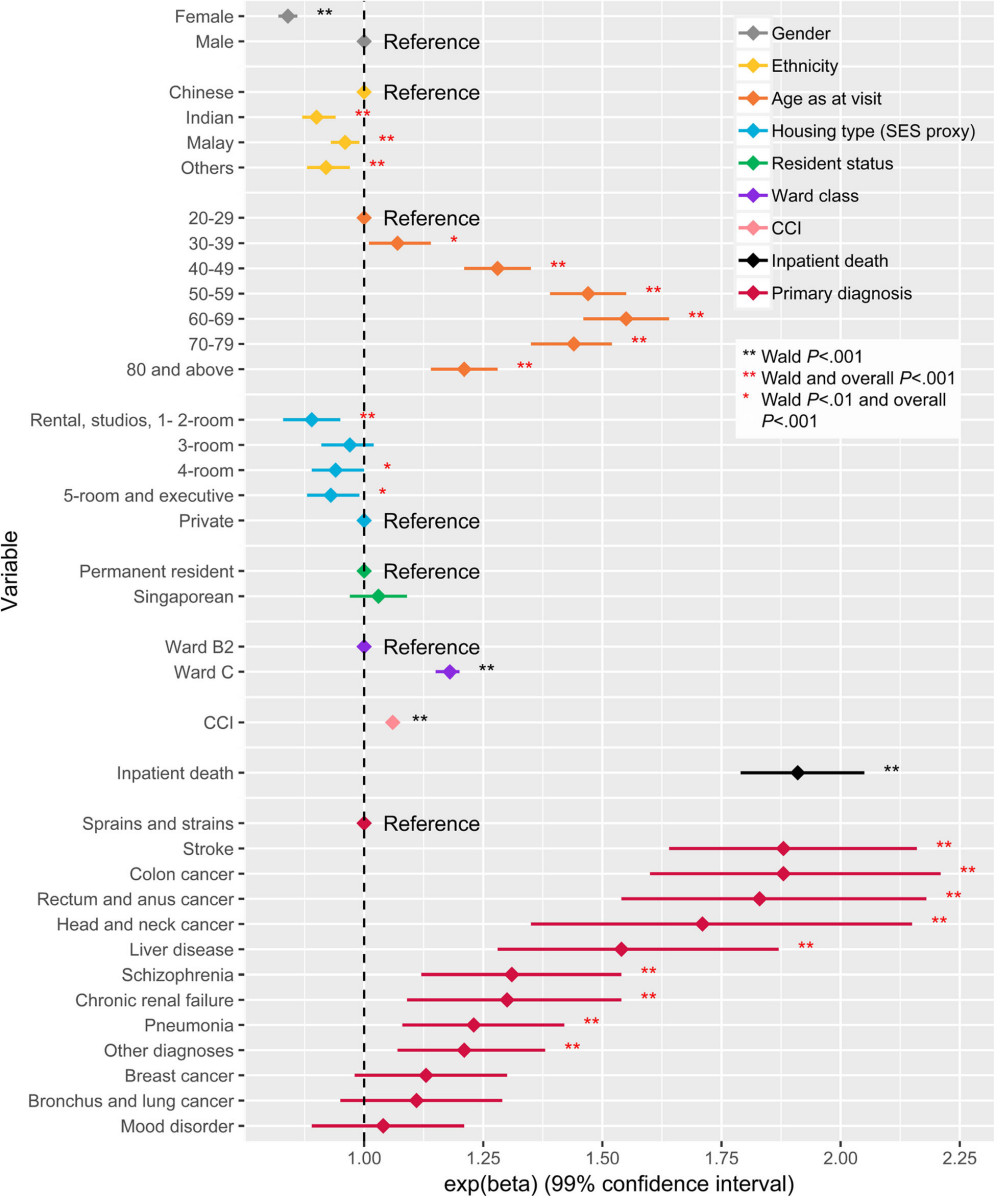
Visualization of the effects of visit factors on expenditure per inpatient visit (expenditure (V)). The diamond corresponds to the estimated effect and the horizontal line corresponds to the 99% confidence interval

The expenditure (V) model showed that gender, ethnicity, age, housing type, ward class, CCI, inpatient death and PD were significantly associated with expenditure (V) with large dynamic ranges in their effects (Additional file 3). Large effects were observed for gender, age, ward class, inpatient death and PD when compared with the other variables in the model. Females had 16% (99% CI: 14–18%) lower expenditure (V) when compared with males. As compared to the youngest group, patients in the older age groups were expected to have higher expenditure (V) and this increase plateaued at the two oldest age groups (Fig. 4). For instance, the 60–69 age group had 55% (99% CI: 46–64%) higher expenditure (V) while the oldest age group had 21% (99% CI: 14–28%) higher expenditure (V) when compared to the youngest age group. Visits in ward C, a ward with more subsidies, had 18% (99% CI: 15–20%) higher expenditure (V) when compared to visits in ward B2. Visits which ended in death had 91% (99% CI: 79–105%) higher expenditure (V). Inpatient visits that had PD of stroke, colon cancer, rectum and anus cancer, head and neck cancer or liver disease were associated with at least 50% higher expenditure (V) when compared to inpatient visits that had PD of sprains and strains.

**Fig. 5:**
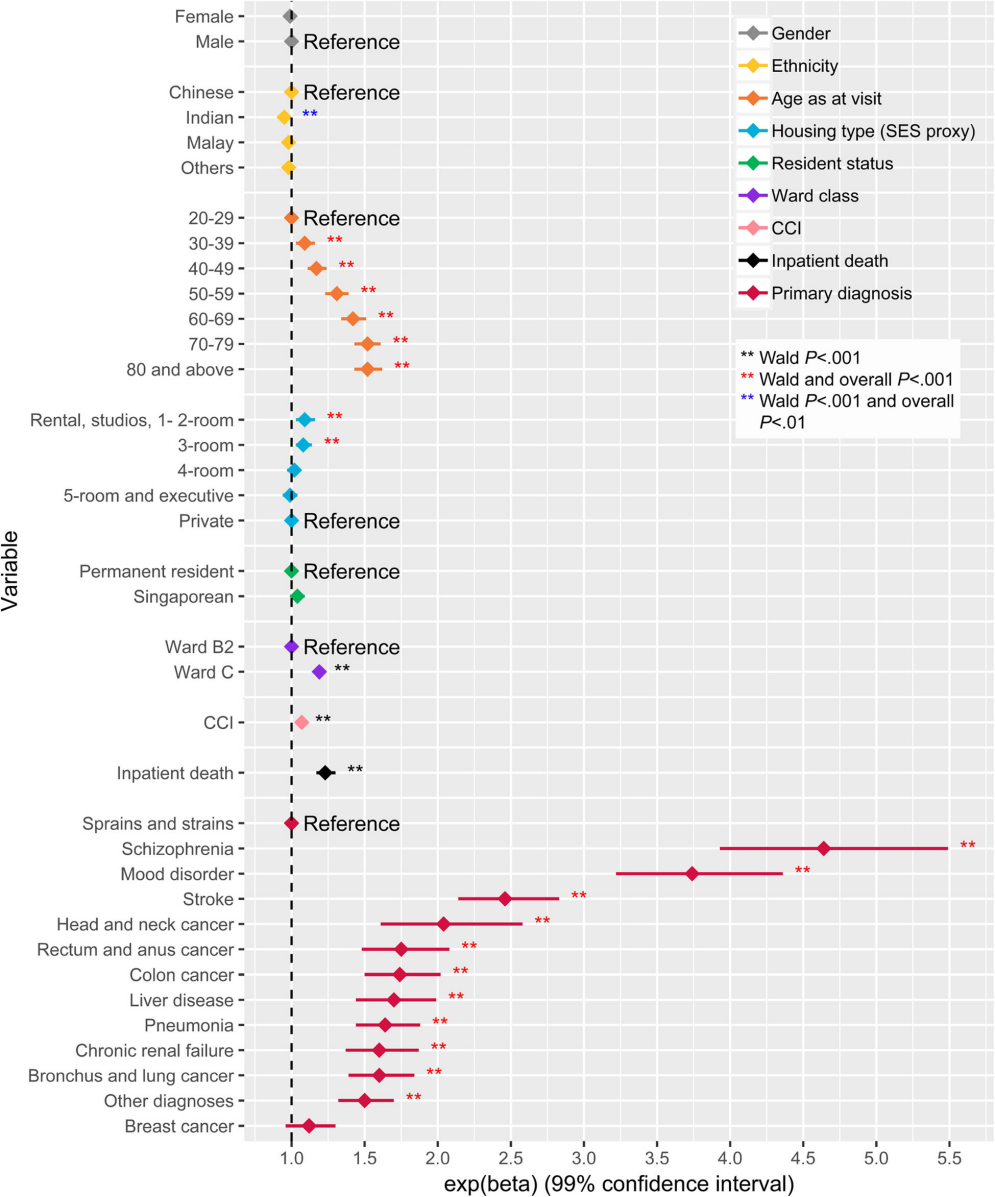
Visualization of the effects of visit factors on length of stay per inpatient visit (LOS (V)). The diamond corresponds to the estimated effect and the horizontal line corresponds to the 99% confidence interval

The LOS (V) model showed that ethnicity, age, housing type, ward class, CCI, inpatient death and PD were significantly associated with LOS (V) with substantial variation in their effects (Additional file 4). Large effects were observed for age, ward class, inpatient death and PD when compared to other variables in the model. When compared to the youngest age group, visits from patients in the older age groups were expected to have higher LOS (V), with age effect increasing monotonically from the youngest to oldest age group, with the two oldest age groups sharing the same effect size (Fig. 5). Visits in ward C, a ward with more subsidy, had 19% (99% CI: 17–21%) higher LOS (V) when compared to visits in ward B2. Visits which ended in death had 23% (99% CI: 17–30%) higher LOS (V). Inpatient visits that had PD of schizophrenia, mood disorders, stroke or head and neck cancer were associated with at least 100% higher LOS (V) when compared to inpatient visits that had PD of sprains and strains.

### Post-hoc and subgroup analyses

Post-hoc analysis showed high correlation between expenditure and LOS (*ρ* = 0.86, *P* < .001). In addition, 74% of total expenditure was also found to be attributed to inpatient visits. Subgroup analysis was performed to better understand the frequency of intensive care among elderly patients. The proportion of inpatient visits which involved ICU or high dependency (HD) admission increased from 6% in the youngest age group to 21% in the 60–69 age group. The proportions declined to 17 and 12% in the 70–79 and 80 and above age groups respectively. Similarly, 41% of inpatient visits in the 21–69 age group involved at least a surgical procedure, compared to 29 and 19% in the 70–79 and 80 and above age groups respectively. Subgroup analyses, examining the relationship between number of inpatient visits and housing type (SES proxy), found that that the average number of inpatient visits per patient decreases as SES increases (rental, studios, 1–2-room: 2.5; 3-room: 2.2; 4-room: 2.1; 5-room and executive: 2.0; private: 1.8). Among visits which ended in death, subgroup analyses showed that its expenditure accounted for 49% of total observed expenditure of a patient on average. Supporting this finding, the analyses showed that 40% of inpatient visits which ended in death involved ICU or HD admissions, compared to just 15% in inpatient visits which did not end in death. This prevalence decreased with age, from 85% in the youngest age group to 24% in the oldest age group.

## Discussion

Increasing age was generally associated with higher expenditure and LOS both at the patient-level and visit-level. However, expenditure plateaued in the highest age groups at both levels, whereas LOS did not. This is despite the high proportion of total expenditure being attributed to inpatient visits and the high correlation between expenditure and LOS. The trend observed in expenditure is likely due to the less intensive and costly treatments in the oldest age groups, consistent with other studies [46–49]. Subgroup analyses supported this hypothesis; ICU and HD admissions, which are costlier than regular admissions, as well as surgical procedures, were less common in the oldest groups of patients. The consistently positive relationship between LOS and age may be driven by factors such as frailty, poor functional status and lack of social support, which are more prevalent with increasing age and are known to be associated with higher LOS [50–52]. Comparing to the limited number of micro level studies that were based on the general patient population, we found that most studies reported non-significant relationship between age and LOS [23, 24], with one of the studies attributing the observation to the suppressor effect caused by other correlated variables [23]. There was however a study which showed positive relationship between age and expenditure with tapering effect in oldest age groups not observed because age was treated as a quantitative variable in the study [25]. Further studies to determine the mechanism and causality could be conducted in the future to better understand this effect.

Patients of lower SES had higher patient expenditure and LOS controlling for all other factors in the models (Figs. 2 and 3). However, at visit-level, such an association is not observed, with patients across most SES groups having relatively similar inpatient expenditure and LOS per inpatient visit. In fact, inpatient visits by patients from lower SES groups had lower expenditure than visits by patients from the highest SES group. This difference observed in the patient- and visit-level models could be due to patients of lower SES groups having more frequent visits resulting in higher accumulated expenditure and LOS over time despite lower per visit expenditure. Our subgroup analyses supported this hypothesis, showing that the average number of inpatient visits per patient decreases as SES increases. Other studies in Singapore have found that the lowest SES group tended to have a longer LOS at index admission, and more frequent inpatient and ED visits [53, 54]. An extensive study of 101 AMCs in the USA also found that frequently admitted patients were likely to be of lower SES [55]. Similar association between lower SES and higher risk of readmission has also been observed in a systematic review based on an elderly population residing in Ireland, Norway, Switzerland, the USA, Canada, Australia, New Zealand and European Union countries [56]. Our finding of a larger cumulative expenditure in this group is concerning as they are least likely to be able to finance their healthcare needs and have lower health literacy [57]. These may further deter them from seeking medical help early due to anxiety regarding affordability [58, 59] and difficulty navigating the healthcare system [60]. Our results call for sufficient integrated safety nets for the low SES groups to cope with care burden as care burden in low SES groups has been shown to affect household structure, mobility, and utilization of social services [61]. Current interventions for frequent admitters in Singapore mostly focus on appropriate-siting of care and redirecting care to the community [62]. Our finding of frequent inpatient visits among the low SES groups indicates that there may be a need to incorporate non-medical support systems in the interventions to address the possible social factors, such as inadequate home amenities for recuperation [63], financial barriers to execute their discharge care plans [64], and lack of transportation for follow-up care [65], that may lead to preventable readmissions [66]. The association between SES and readmission also highlights the importance of factoring SES in the formulation of risk scores for readmission and frequent admissions. Further studies are needed to explore reasons for longer total LOS, more frequent inpatient visits and higher total expenditure in the lower SES groups, despite demographics and medical complexity being similar.

Inpatient visits that ended in death were expected to cost 91% more after controlling for the other factors. Expenditure of visit which ended in death accounted for almost half of total observed expenditure of a patient on average. Although analysis was performed at patient-level, a study in India found that 2014 to 2015 inpatient expenditure for decedents was 64% higher than non-decedent [67]. This observation may be due to more intensive care rendered at the end-of-life (EOL), prior to the patient passing on in the hospital. This finding has been reported in other studies [68, 69]. Supporting this observation, subgroup analyses showed that ICU and HD admissions were almost three times more common in visits which ended in death than in those which did not end in death, with the prevalence decreasing with age. While intensive EOL care is sometimes viewed as a waste of resources, often it is not possible to determine prospectively whether treatment is life-saving or futile, and it would be too simplistic to classify this observation as inappropriate deployment of resources [70]. Further studies to examine the cost, type and appropriateness of treatment at the EOL are needed to better understand these observations, to inform policies and interventions in the EOL group.

There were also evidences of positive associations between staying in a more heavily subsidized ward with higher expenditure and LOS per inpatient visit. Given that the analysis had accounted for SES, the positive associations were most likely a by-product of the difference in cost. As the study is not qualitative in nature, we are unable to ascertain the underlying decision making process of the patients during their inpatient stays. However, there are several possible cost-motivated reasons which could have led to this observation. The observation could be a reflection of consumer behavior in response to lower OOP payments when admitted to wards with higher subsidies, similar to effect of insurance on utilization, where patients with generous insurance coverage were expected to have higher utilization [71]. As patients are given financial counselling to allow them to make informed decision of ward class based on preference and budget before hospitalization, the observation could reflect patients’ decision to opt for a more heavily subsidized ward class in anticipation of longer LOS. It could also be due to patients downgrading to a more heavily subsidized ward class half-way in their hospitalization as the LOS and expenditure increases. To the best of our knowledge, there is paucity of studies investigating the effect of lower OOP from governmental subsidies on utilization. At meso level, growth in total healthcare expenditure at national level has been reported in the USA after patient cost-sharing was introduced [72]. This finding expands on current understanding of OOP payments and consumption in terms of expenditure and LOS and has significant implications on how health systems, insurers and governments structure their fees, coverage and subsidies respectively. If the increase in expenditure and LOS per inpatient visit was motivated by the difference in cost, there is a need for the public health system to investigate whether the amount of subsidy provided is above the optimum level. Qualitative studies are needed to better understand how patients factor in financial cost in their healthcare decisions.

Our analyses showed the conditions that drove expenditure and LOS were largely similar. A history of PD of cancers, renal diseases, cardiovascular diseases were highly associated with increased expenditure and LOS per patient. In addition, a history of mental illnesses was highly associated with increased LOS per patient, disproportionately so when compared to its association with expenditure. At the visit-level, these associations largely held true as well, and discrepancies between LOS and expenditure were also observed. Of note, visits with PD of mood disorder were not expected to cost more than the reference group despite its strong association with per visit LOS. These admissions might be more social than medical in nature, resulting in a lower treatment (and hence per visit) expenditure in keeping with our findings at the patient-level [73]. These findings suggest the importance of examining multiple utilization metrics, given the different condition drivers for each resource category, and the need to optimize and plan for different types of resources.

Strengths of this study include the large cohort size, long study duration and the comprehensive adjustment for common and high cost diagnoses in investigation of drivers of expenditure and utilization (in terms of LOS). The results from this study also have greater generalizability as we used hospital-wide data rather than insurance claims data, where the latter may contain incomplete records of utilization and have more volatile patient populations [74]. Two levels of analyses were performed in our study, providing insights on both short-term and long-term drivers of hospital utilization. However, we acknowledge that the patient models were not able to capture temporality and completely account for comorbidity. Including comorbidity score as a variable in the patient models will lead to a counterintuitive interpretation due to the inter-relationship between PD and the comorbidity score. As the comorbidity score is derived from presence of conditions that are already accounted for in the models, the interpretation that two patients have the same comorbidity scores but with different values for a particular medical condition in the model is not valid. Also, as it was not feasible to include all individual PD in the model due to the large number of conditions documented in the data, we chose to focus on the top conditions found in the patient models. In addition, there could be potential selection bias from the exclusion of patients and inpatient visits with missing variables. Majority of the patients and inpatient visits were excluded due to no PD mapped by CCS at patient-level, missing SES at patient-level and missing PD at visit-level. Some differences in characteristics were observed between the excluded patients and inpatient visits and the analyzed samples (Additional files 5, 6 and 7). These differences suggest that there could be over- and under-estimation of the effects in the patient and visit models. However, with a missing rate of 16% out of the total subsidized patients and 8% out of the total subsidized inpatient visits, and a distinct characteristic of patients with missing SES (i.e. PRs) corresponding to the patient minority in the main analysis, such bias is likely to be modest [75].

As with many health systems globally, data across health systems is not linked, hence the analyses were based on a single AMC within Singapore’s NUHS. However, despite this limitation, the findings from a single hospital can still be applicable to other hospitals that serve a similar population. Our results are generalizable to other hospitals in Singapore as there are no systematic differences in the population that the different health systems serve. Furthermore, the set up and structure of each health system in Singapore is similar in nature. A recent study comparing the different health systems in Singapore showed that the patient populations served by each have a similar age distribution and mean number of chronic diseases [76]. At an international level, the challenges that face healthcare systems in developed nations are similar to those examined in this study – an ageing population with increased chronic disease burden, and a resultant increase in healthcare spending that threatens the sustainability of healthcare systems. While there are differences between the populations and healthcare systems of each country, the approach and the drivers here, could serve as a useful starting point and comparison with other countries. Moreover, based on our results, we see that amongst those variables (e.g. age, SES, inpatient death) that have been studied elsewhere, our results have corroborated well with those studies. For example, our observation of less costly and less intensive care in the oldest age groups [46–49], association between lower SES with increased frequency of inpatient visits [53–56], and excessive inpatient expenditure from decedents [67] have also been reported in other studies. These give us confidence that the novel results in our studies could apply to those populations and countries as well.

Due to unavailability of latest data at the point of analysis, the study only included data from 2005 to 2013. However, given the relatively long time horizon of the data and the fact that we are analyzing trends across time, this is less likely to pose a challenge to the interpretation of the results. Investigating whether the associations between the examined variables hold true in data after 2013 could be an area of future research.

Nevertheless, these findings add to the growing body of literature on healthcare utilization and may be useful for policy makers and fellow health services researchers to understand factors associated with hospital expenditure and LOS and aid the formulation of future policies, interventions and research.

## Conclusions

Demographics, SES, PD and observed period were associated with expenditure and LOS at patient-level. Demographics, SES, ward class, comorbidity score, inpatient death and PD were associated with expenditure and LOS at visit-level. Although adjusted expenditure and LOS per inpatient visit were largely similar across SES groups, patients of lower SES accumulated greater expenditure and LOS over time due to more frequent visits. We found evidences of positive association of staying in a more heavily subsidized ward with expenditure and LOS per inpatient visit, adjusted for SES, possibly reflecting patients’ cost-motivated consumer behavior. Inpatient death was highly associated with increased expenditure for that inpatient visit. Conditions that drove expenditure and LOS were largely similar, with mental illnesses disproportionately affecting LOS, suggesting the importance of examining multiple utilization metrics to better optimize and plan for different types of resources based on their different drivers. Findings from this study will inform health policy makers, professionals and administrators in identifying target areas for policy and service planning for management of expenditure and resource use.

## Additional files

Additional file 1: The effects of patient factors on expenditure per patient (expenditure (P)) (DOCX 16 kb)

Additional file 2: The effects of patient factors on length of stay per patient (LOS (P)) (DOCX 16 kb)

Additional file 3: The effects of visit factors on expenditure per inpatient visit (expenditure (V)) (DOCX 16 kb)

Additional file 4: The effects of visit factors on length of stay per inpatient visit (LOS (V)) (DOCX 16 kb)

Additional file 5: Summary of patient and visit factors of patients who had no primary diagnosis mapped by Clinical Classification Software (DOCX 15 kb)

Additional file 6: Summary of patient and visit factors of patient who had missing housing type (socio-economic status proxy) (DOCX 15 kb)

Additional file 7: Summary of visit factors of 5779 inpatient visits which had missing primary diagnosis (DOCX 14 kb)

## Abbreviations

AMC: academic medical centre
CCI: charlson comorbidity index
CCS: clinical classification software
CI: confidence interval
ED: emergency department
EMR: electronic medical records
EOL: end-of-life
GDP: gross domestic product
GEE: generalized estimating equations
GLM: generalized linear model
GVIF: generalized variance-inflation factor
HD: high dependency
ICU: intensive care unit
LOS: length of stay
NUH: national university hospital
NUHS: national university health system
OECD: organisation for economic co-operation and development
OOP: out-of-pocket
PD: primary diagnosis
PR: permanent resident
SES: socio-economic status
SOC: specialist outpatient clinic
USA: united states of america
VIF: variance-inflation factor
