# Supplementary material for: Drivers of hospital expenditure and length of stay in an academic medical centre: a retrospective cross-sectional study
Source: BMC Health Serv Res. 2019 Jul 2;19:442. doi: 10.1186/s12913-019-4248-1 (PMC6604431; doi:10.1186/s12913-019-4248-1)
Supplement: Supplementary file 1 — The effects of patient factors on expenditure per patient (expenditure (P)) (DOCX 16 kb) [file 12913_2019_4248_MOESM1_ESM.docx]

**Additional file 1:** **The effects of patient factors on expenditure per patient (expenditure (P))**

| Variable | exp(β) | 99% CI | | Wald test *P* | Overall *P* |
| --- | --- | --- | --- | --- | --- |
|  |  | Lower | Upper |  |  |
| Gender |  |  |  |  |  |
| Female | 0.97 | 0.94 | 1.00 | .026 |  |
| Male | 1.00 | - | - | - |  |
| Ethnicity |  |  |  |  | <.001 |
| Chinese | 1.00 | - | - | - |  |
| Indian | 0.99 | 0.94 | 1.05 | .700 |  |
| Malay | 1.11 | 1.06 | 1.16 | <.001 |  |
| Others | 0.91 | 0.86 | 0.97 | <.001 |  |
| Age as at first contact |  |  |  |  | <.001 |
| 21-29 | 1.00 | - | - | - |  |
| 30-39 | 1.27 | 1.21 | 1.33 | <.001 |  |
| 40-49 | 1.72 | 1.64 | 1.80 | <.001 |  |
| 50-59 | 2.30 | 2.19 | 2.41 | <.001 |  |
| 60-69 | 3.03 | 2.86 | 3.21 | <.001 |  |
| 70-79 | 3.74 | 3.50 | 3.99 | <.001 |  |
| 80 and above | 3.65 | 3.35 | 4.00 | <.001 |  |
| Housing type (socio-economic status proxy) |  |  |  |  |  |
| Rental, studios, 1- 2-room | 1.80 | 1.65 | 1.97 | <.001 | <.001 |
| 3-room | 1.65 | 1.56 | 1.75 | <.001 |  |
| 4-room | 1.52 | 1.44 | 1.61 | <.001 |  |
| 5-room and executive | 1.41 | 1.33 | 1.49 | <.001 |  |
| Private | 1.00 | - | - | - |  |
| Resident status |  |  |  |  |  |
| Permanent resident | 1.00 | - | - | - |  |
| Singaporean | 1.76 | 1.68 | 1.84 | <.001 |  |
| Primary diagnosis |  |  |  |  |  |
| Chronic renal failure | 5.27 | 4.45 | 6.31 | <.001 |  |
| Breast cancer | 5.08 | 4.28 | 6.10 | <.001 |  |
| Head and neck cancer | 4.66 | 3.45 | 6.52 | <.001 |  |
| Liver disease | 4.33 | 3.60 | 5.28 | <.001 |  |
| Bronchus and lung cancer | 3.96 | 3.23 | 4.95 | <.001 |  |
| Colon cancer | 3.63 | 2.88 | 4.63 | <.001 |  |
| Stroke | 3.02 | 2.76 | 3.32 | <.001 |  |
| Rectum and anus cancer | 2.81 | 2.14 | 3.77 | <.001 |  |
| Pneumonia | 2.77 | 2.53 | 3.03 | <.001 |  |
| Schizophrenia | 2.68 | 2.09 | 3.51 | <.001 |  |
| Acute myocardial infarction | 2.55 | 2.32 | 2.81 | <.001 |  |
| Coronary heart disease | 2.44 | 2.24 | 2.65 | <.001 |  |
| Osteoarthritis | 2.38 | 2.03 | 2.82 | <.001 |  |
| Hepatitis | 2.27 | 1.78 | 2.96 | <.001 |  |
| Diabetes mellitus with complication | 2.13 | 1.81 | 2.52 | <.001 |  |
| Acute renal failure | 2.11 | 1.69 | 2.68 | <.001 |  |
| Gastroduodenal ulcer | 2.05 | 1.73 | 2.44 | <.001 |  |
| Paralysis | 1.98 | 1.62 | 2.47 | <.001 |  |
| Congestive heart failure | 1.88 | 1.67 | 2.12 | <.001 |  |
| Diabetes mellitus without complication | 1.87 | 1.65 | 2.13 | <.001 |  |
| Urinary tract infection | 1.83 | 1.69 | 1.99 | <.001 |  |
| Mood disorder | 1.80 | 1.53 | 2.14 | <.001 |  |
| Dementia | 1.69 | 1.38 | 2.11 | <.001 |  |
| Chronic obstructive pulmonary disease | 1.60 | 1.40 | 1.85 | <.001 |  |
| Esophageal disorder | 1.52 | 1.32 | 1.77 | <.001 |  |
| Spondylosis | 1.52 | 1.40 | 1.67 | <.001 |  |
| Hyperlipidemia | 1.39 | 1.17 | 1.67 | <.001 |  |
| Gout and other crystal arthropathy | 1.28 | 1.06 | 1.56 | <.01 |  |
| Hypertension | 1.26 | 1.15 | 1.37 | <.001 |  |
| Observed period | 1.05 | 1.04 | 1.05 | <.001 |  |
