# Supplementary material for: Drivers of hospital expenditure and length of stay in an academic medical centre: a retrospective cross-sectional study
Source: BMC Health Serv Res. 2019 Jul 2;19:442. doi: 10.1186/s12913-019-4248-1 (PMC6604431; doi:10.1186/s12913-019-4248-1)
Supplement: Supplementary file 2 — The effects of patient factors on length of stay per patient (LOS (P)) (DOCX 16 kb) [file 12913_2019_4248_MOESM2_ESM.docx]

**Additional file 2:** **The effects of patient factors on length of stay per patient (LOS (P))**

| Variable | exp(β) | 99% CI | | Wald test *P* | Overall *P* |
| --- | --- | --- | --- | --- | --- |
|  |  | Lower | Upper |  |  |
| Gender |  |  |  |  |  |
| Female | 1.09 | 1.06 | 1.11 | <.001 |  |
| Male | 1.00 | - | - | - |  |
| Ethnicity |  |  |  |  |  |
| Chinese | 1.00 | - | - | - | <.001 |
| Indian | 1.16 | 1.11 | 1.20 | <.001 |  |
| Malay | 1.38 | 1.34 | 1.42 | <.001 |  |
| Others | 1.13 | 1.08 | 1.18 | <.001 |  |
| Age as at first contact |  |  |  |  | <.001 |
| 21-29 | 1.00 | - | - | - |  |
| 30-39 | 1.42 | 1.37 | 1.47 | <.001 |  |
| 40-49 | 1.89 | 1.82 | 1.96 | <.001 |  |
| 50-59 | 2.69 | 2.59 | 2.79 | <.001 |  |
| 60-69 | 4.00 | 3.83 | 4.17 | <.001 |  |
| 70-79 | 6.10 | 5.81 | 6.41 | <.001 |  |
| 80 and above | 7.91 | 7.42 | 8.43 | <.001 |  |
| Housing type (socio-economic status proxy) |  |  |  |  | <.001 |
| Rental, studios, 1- 2-room | 3.06 | 2.86 | 3.27 | <.001 |  |
| 3-room | 2.35 | 2.24 | 2.46 | <.001 |  |
| 4-room | 1.97 | 1.88 | 2.05 | <.001 |  |
| 5-room and executive | 1.68 | 1.60 | 1.75 | <.001 |  |
| Private | 1.00 | - | - | - |  |
| Resident status |  |  |  |  |  |
| Permanent resident | 1.00 | - | - | - |  |
| Singaporean | 1.78 | 1.72 | 1.85 | <.001 |  |
| Primary diagnosis |  |  |  |  |  |
| Schizophrenia | 7.30 | 6.10 | 8.82 | <.001 |  |
| Liver disease | 5.29 | 4.63 | 6.08 | <.001 |  |
| Chronic renal failure | 5.18 | 4.58 | 5.87 | <.001 |  |
| Bronchus and lung cancer | 4.94 | 4.26 | 5.77 | <.001 |  |
| Mood disorder | 4.90 | 4.36 | 5.53 | <.001 |  |
| Head and neck cancer | 4.83 | 3.88 | 6.11 | <.001 |  |
| Stroke | 4.52 | 4.23 | 4.83 | <.001 |  |
| Pneumonia | 3.75 | 3.52 | 4.00 | <.001 |  |
| Colon cancer | 3.47 | 2.95 | 4.12 | <.001 |  |
| Breast cancer | 3.05 | 2.69 | 3.47 | <.001 |  |
| Diabetes mellitus with complication | 3.00 | 2.67 | 3.38 | <.001 |  |
| Rectum and anus cancer | 2.80 | 2.30 | 3.44 | <.001 |  |
| Paralysis | 2.63 | 2.27 | 3.07 | <.001 |  |
| Urinary tract infection | 2.61 | 2.46 | 2.77 | <.001 |  |
| Acute renal failure | 2.60 | 2.21 | 3.08 | <.001 |  |
| Congestive heart failure | 2.56 | 2.35 | 2.79 | <.001 |  |
| Dementia | 2.46 | 2.13 | 2.87 | <.001 |  |
| Coronary heart disease | 2.26 | 2.13 | 2.40 | <.001 |  |
| Gastroduodenal ulcer | 2.25 | 1.99 | 2.55 | <.001 |  |
| Hepatitis | 2.20 | 1.84 | 2.65 | <.001 |  |
| Osteoarthritis | 2.10 | 1.87 | 2.37 | <.001 |  |
| Acute myocardial infarction | 2.05 | 1.92 | 2.20 | <.001 |  |
| Chronic obstructive pulmonary disease | 2.04 | 1.85 | 2.26 | <.001 |  |
| Diabetes mellitus without complication | 1.97 | 1.80 | 2.16 | <.001 |  |
| Gout and other crystal arthropathy | 1.68 | 1.46 | 1.93 | <.001 |  |
| Spondylosis | 1.60 | 1.50 | 1.70 | <.001 |  |
| Esophageal disorders | 1.45 | 1.31 | 1.62 | <.001 |  |
| Hyperlipidemia | 1.32 | 1.16 | 1.51 | <.001 |  |
| Hypertension | 1.12 | 1.06 | 1.20 | <.001 |  |
| Observed period | 1.04 | 1.03 | 1.04 | <.001 |  |
