# Supplementary material for: Drivers of hospital expenditure and length of stay in an academic medical centre: a retrospective cross-sectional study
Source: BMC Health Serv Res. 2019 Jul 2;19:442. doi: 10.1186/s12913-019-4248-1 (PMC6604431; doi:10.1186/s12913-019-4248-1)
Supplement: Supplementary file 4 — The effects of visit factors on length of stay per inpatient visit (LOS (V)) (DOCX 16 kb) [file 12913_2019_4248_MOESM4_ESM.docx]

**Additional file 4:** **The effects of visit factors on length of stay per inpatient visit (LOS (V))**

| Variable | exp(β) | 99% CI | | Wald test *P* | Overall *P* |
| --- | --- | --- | --- | --- | --- |
|  |  | Lower | Upper |  |  |
| Gender |  |  |  |  |  |
| Female | 0.99 | 0.96 | 1.01 | .128 |  |
| Male | 1.00 | - | - | - |  |
| Ethnicity |  |  |  |  | <.01 |
| Chinese | 1.00 | - | - | - |  |
| Indian | 0.95 | 0.91 | 0.99 | <.001 |  |
| Malay | 0.98 | 0.96 | 1.01 | .120 |  |
| Others | 0.98 | 0.94 | 1.02 | .120 |  |
| Age as at visit |  |  |  |  | <.001 |
| 21-29 | 1.00 | - | - | - |  |
| 30-39 | 1.09 | 1.03 | 1.16 | <.001 |  |
| 40-49 | 1.17 | 1.11 | 1.24 | <.001 |  |
| 50-59 | 1.31 | 1.23 | 1.39 | <.001 |  |
| 60-69 | 1.42 | 1.34 | 1.51 | <.001 |  |
| 70-79 | 1.52 | 1.43 | 1.61 | <.001 |  |
| 80 and above | 1.52 | 1.43 | 1.62 | <.001 |  |
| Housing type (SES proxy) |  |  |  |  | <.001 |
| Rental, studios, 1- 2-room | 1.09 | 1.03 | 1.16 | <.001 |  |
| 3-room | 1.08 | 1.03 | 1.14 | <.001 |  |
| 4-room | 1.02 | 0.97 | 1.06 | .428 |  |
| 5-room and executive | 0.99 | 0.94 | 1.04 | .686 |  |
| Private | 1.00 | - | - | - |  |
| Resident status |  |  |  |  |  |
| Permanent resident | 1.00 | - | - | - |  |
| Singaporean | 1.04 | 0.99 | 1.09 | .024 |  |
| Ward class |  |  |  |  |  |
| Ward B2 | 1.00 | - | - | - |  |
| Ward C | 1.19 | 1.17 | 1.21 | <.001 |  |
| CCI | 1.07 | 1.07 | 1.08 | <.001 |  |
| Inpatient death | 1.23 | 1.17 | 1.30 | <.001 |  |
| Primary diagnosis |  |  |  |  | <.001 |
| Sprains and strains | 1.00 | - | - | - |  |
| Schizophrenia | 4.64 | 3.93 | 5.49 | <.001 |  |
| Mood disorder | 3.74 | 3.22 | 4.36 | <.001 |  |
| Stroke | 2.46 | 2.14 | 2.83 | <.001 |  |
| Head and neck cancer | 2.04 | 1.61 | 2.58 | <.001 |  |
| Rectum and anus cancer | 1.75 | 1.48 | 2.08 | <.001 |  |
| Colon cancer | 1.74 | 1.50 | 2.02 | <.001 |  |
| Liver disease | 1.70 | 1.44 | 1.99 | <.001 |  |
| Pneumonia | 1.64 | 1.44 | 1.88 | <.001 |  |
| Chronic renal failure | 1.60 | 1.37 | 1.87 | <.001 |  |
| Bronchus and lung cancer | 1.60 | 1.39 | 1.84 | <.001 |  |
| Others | 1.50 | 1.32 | 1.70 | <.001 |  |
| Breast cancer | 1.12 | 0.96 | 1.30 | .051 |  |
