# Supplementary material for: Drivers of hospital expenditure and length of stay in an academic medical centre: a retrospective cross-sectional study
Source: BMC Health Serv Res. 2019 Jul 2;19:442. doi: 10.1186/s12913-019-4248-1 (PMC6604431; doi:10.1186/s12913-019-4248-1)
Supplement: Supplementary file 5 — Summary of patient and visit factors of patients who had no primary diagnosis mapped by Clinical Classification Software (DOCX 15 kb) [file 12913_2019_4248_MOESM5_ESM.docx]

**Additional file 5:** **Summary of patient and visit factors of patients who had no primary diagnosis mapped by Clinical Classification Software**

| Categorical variable | Frequency (%) | |
| --- | --- | --- |
|  | By patient (N = 1746) | By inpatient visit (n = 220) |
| Female | 924 (52.9) | 111 (50.5) |
| Ethnicity |  |  |
| Chinese | 1326 (75.9) | 181 (82.3) |
| Indian | 125 (7.2) | 16 (7.3) |
| Malay | 186 (10.7) | 14 (6.4) |
| Others | 109 (6.2) | 9 (4.1) |
| Age^a^ |  |  |
| 21-29 | 376 (21.5) | 38 (17.3) |
| 30-39 | 272 (15.6) | 19 (8.6) |
| 40-49 | 351 (20.1) | 32 (14.5) |
| 50-59 | 366 (21.0) | 56 (25.5) |
| 60-69 | 251 (14.4) | 46 (20.9) |
| 70-79 | 100 (5.7) | 20 (9.1) |
| 80 and above | 30 (1.7) | 9 (4.1) |
| Housing type (socio-economic status proxy) |  |  |
| Rental, studios, 1- 2-room | 30 (1.7) | 8 (3.6) |
| 3-room | 362 (20.7) | 50 (22.7) |
| 4-room | 600 (34.4) | 85 (38.6) |
| 5-room | 454 (26.0) | 49 (22.3) |
| Private | 183 (10.5) | 14 (6.4) |
| NA | 117 (6.7) | 14 (6.4) |
| Singaporean | 1534 (87.9) | 206 (93.6) |
| Inpatient death | 1 (0.1) | 1 (0.5) |
| ^a^Refers to age as at first contact for patient-level and age as at visit for visit-level | | |

| Numerical variable | Total (median; interquartile range) | |
| --- | --- | --- |
|  | By patient (N = 1746) | By inpatient visit (n = 220) |
| Hospital expenditure | 6,298,619 (1331; 460-4068) | 2,367,245 (7694; 5172-9115) |
| Length of stay | 1002 (0; 0-0) | 1002 (2; 2-2) |
| Inpatient visits | 220 (0; 0-0) |  |
| CCI | (0; 0-0) | (0; 0-1) |
| Observed period (years) | (4; 2-7) |  |
