# Supplementary material for: Drivers of hospital expenditure and length of stay in an academic medical centre: a retrospective cross-sectional study
Source: BMC Health Serv Res. 2019 Jul 2;19:442. doi: 10.1186/s12913-019-4248-1 (PMC6604431; doi:10.1186/s12913-019-4248-1)
Supplement: Supplementary file 6 — Summary of patient and visit factors of patient who had missing housing type (socio-economic status proxy) (DOCX 15 kb) [file 12913_2019_4248_MOESM6_ESM.docx]

**Additional file 6: Summary of patient and visit factors of patient who had missing housing type (socio-economic status proxy)**

| Categorical variable | Frequency (%) | |
| --- | --- | --- |
|  | By patient (N = 52,855) | By inpatient visit (n = 12,941) |
| Female | 11,586 (21.9) | 4207 (32.5) |
| Ethnicity |  |  |
| Chinese | 23,605 (44.7) | 7952 (61.4) |
| Indian | 11,546 (21.8) | 2052 (15.9) |
| Malay | 2298 (4.3) | 1829 (14.1) |
| Others | 15,406 (29.1) | 1108 (8.6) |
| Age^a^ |  |  |
| 21-29 | 24,179 (45.7) | 1409 (10.9) |
| 30-39 | 15,163 (28.7) | 1345 (10.4) |
| 40-49 | 6553 (12.4) | 1417 (10.9) |
| 50-59 | 2815 (5.3) | 1824 (14.1) |
| 60-69 | 1754 (3.3) | 1952 (15.1) |
| 70-79 | 1374 (2.6) | 2534 (19.6) |
| 80 and above | 1017 (1.9) | 2460 (19.0) |
| Singaporean | 9882 (18.7) | 10,380 (80.2) |
| Inpatient death | 434 (0.8) | 434 (3.4) |
| ^a^Refers to age as at first contact for patient-level and age as at visit for visit-level | | |

| Numerical variable | Total (median; interquartile range) | |
| --- | --- | --- |
|  | By patient (N = 52,855) | By inpatient visit (n = 12,941) |
| Hospital expenditure | 132,544,146 (264; 221-467) | 93,167,603 (3343; 1707-7270) |
| Length of stay | 93,686 (0; 0-0) | 93,686 (4; 2-7) |
| Inpatient visits | 12,941 (0; 0-0) |  |
| CCI | (0; 0-0) | (1; 0-3) |
| Observed period (years) | (4; 2-6) |  |
