# Supplementary material for: Drivers of hospital expenditure and length of stay in an academic medical centre: a retrospective cross-sectional study
Source: BMC Health Serv Res. 2019 Jul 2;19:442. doi: 10.1186/s12913-019-4248-1 (PMC6604431; doi:10.1186/s12913-019-4248-1)
Supplement: Supplementary file 7 — Summary of visit factors of 5779 inpatient visits which had missing primary diagnosis (DOCX 14 kb) [file 12913_2019_4248_MOESM7_ESM.docx]

**Additional file 7: Summary of visit factors of 5779 inpatient visits which had missing primary diagnosis**

| Categorical variable | Frequency (%) |
| --- | --- |
| Female | 2731 (47.3) |
| Ethnicity |  |
| Chinese | 3513 (60.8) |
| Indian | 519 (9.0) |
| Malay | 1293 (22.4) |
| Others | 454 (7.9) |
| Age as at visit |  |
| 21-29 | 252 (4.4) |
| 30-39 | 251 (4.3) |
| 40-49 | 600 (10.4) |
| 50-59 | 1264 (21.9) |
| 60-69 | 1336 (23.1) |
| 70-79 | 1242 (21.5) |
| 80 and above | 834 (14.4) |
| Housing type (socio-economic status proxy) |  |
| Rental, studios, 1- 2-room | 463 (8.0) |
| 3-room | 1770 (30.6) |
| 4-room | 2033 (35.2) |
| 5-room | 1309 (22.7) |
| Private | 204 (3.5) |
| Singaporean | 5583 (96.6) |
| Inpatient death | 94 (1.6) |

| Numerical variable | Total (median; interquartile range) |
| --- | --- |
| Hospital expenditure | 49,711,105 (3947; 2097-8672) |
| Length of stay | 44,915 (4; 2-8) |
| CCI | (2; 1-5) |
